# Urinary Albumin to Creatinine Ratio to Predict Diabetic Retinopathy: The Eyes Have It!

**DOI:** 10.7759/cureus.22902

**Published:** 2022-03-06

**Authors:** Divya Prabhu, Akshay Rao, Anjana Rajanna, Sakthi Kannan, Selva Kumar

**Affiliations:** 1 Internal Medicine, Ramaiah Medical College, Bangalore, IND; 2 Internal medicine, Ramaiah Medical College, Bangalore, IND

**Keywords:** macroalbuminuria, microalbuminuria, retinopathy screening, fundoscopy, diabetic retniopathy, uacr

## Abstract

Introduction

The Coronavirus disease 2019 (COVID-19) pandemic has provided a push in the search for alternative screening methods to replace annual fundoscopic examination of patients with type 2 diabetes mellitus (T2DM) to detect diabetic retinopathy (DR).

Materials and methods

This retrospective study was conducted using the data of T2DM patients from their routine follow-up hospital visits. The details from their history and physical examination were extracted. As part of their routine follow-up visit, they had undergone a panel of investigations that included blood glucose measurements and urinary albumin excretion measurements. Univariate and logistic multivariate regression analyses were applied to identify the potential clinical and laboratory parameters associated with the presence of DR in them.

Results

Analysis of the medical records of 272 T2DM patients revealed that 147 patients had DR while 125 did not. Furthermore, 135 had non-proliferative DR (64 mild, 53 moderate, and 18 severe grades), whereas the remaining 12 had proliferative DR. On sequential univariate and multiple regression analysis, urinary albumin creatinine ratio (UACR), known duration of T2DM, and history of ischemic heart disease were seen to be independently associated with the presence of DR. Median UACR for those without DR was 42.6 mg/g (range 18.21-183.3 mg/g) while for those with retinopathy it was 214 mg/g (range 45.4-1260 mg/g) (p<0.001). The receiver operating characteristics curve analysis provided an area under the curve of 70% for UACR. UACR value of 140 mg/g could predict the presence of DR with a sensitivity of 60.5% & specificity of 72%, as well as had positive and negative likelihood ratios of 2.16 and 0.54, respectively.

Conclusion

UACR has the potential to be used as a screening tool for DR until the easing of social restrictions due to the COVID-19 pandemic.

## Introduction

Type 2 diabetes mellitus (T2DM) is characterized by microvascular damage mediated by long-standing hyperglycemia. Microvascular complications like diabetic retinopathy (DR), nephropathy, and neuropathy tend to occur as a result of poorly controlled T2DM. These complications contribute greatly to the morbidity and mortality in T2DM. DR is increasingly being recognized as a significant cause of blindness in India as in the rest of the world [1,2]. In advanced stages, it can progress in a relentless manner despite best attempts to achieve optimal blood glucose control [3]. It has been recommended that all patients with T2DM undergo fundoscopic examination at diagnosis and at least annually thereafter for early detection and management of DR [4].

The pandemic of Coronavirus Disease 2019 (COVID-19) has disrupted this practice of routine fundoscopic examination [5]. Initially, the lockdown imposed by the government and subsequently the fear of COVID-19 exposure at hospitals may have deterred patients from adhering to their ophthalmologist appointment. In the midst of the deadly pandemic, when the healthcare services were stretched to their limits, screening of asymptomatic patients for retinal findings was accorded a lower priority. During fundus examination, the patient and the examiner are separated by virtually no distance. The risk of close contact with patients carrying potential subclinical COVID-19 infection might have also led to some degree of reluctance among the medical fraternity to perform fundoscopy for retinopathy screening. Indeed it has been acknowledged that ophthalmologists are at a higher risk of being infected with COVID-19 [6]. Consequently, this has spurred the search for alternative markers that can be used to screen T2DM patients for the need to undergo fundus examination.

The association between diabetic nephropathy and retinopathy in T2DM is not as strong as in the case of type 1 diabetes. This might be due to the presence of other contributors to renal injury in T2DM, such as associated essential hypertension, dyslipidemia, and atherosclerosis [7]. Yet one underlying mechanism that is common to the pathogenesis of both DR and diabetic nephropathy is endothelial dysfunction [8]. The histopathological hallmark of DR is a loss of pericytes which are cells that cover the retinal capillary endothelial cells and help to maintain the capillary tone. This is followed by thickening of the basement membrane and enhanced endothelial permeability. Identical histological changes are known to occur in the kidney within the glomeruli of nephrons which are the tuft of capillaries enclosed by the Bowman’s capsule. This leads to an increase in endothelial permeability resulting in albuminuria [9,10].

The determination of the urinary albumin to creatinine ratio (UACR) using spot, first-morning urine sample is a simple and accurate test to quantify albuminuria [11]. All patients with T2DM are recommended to test UACR levels annually in order to detect the occurrence of nephropathy [4]. Hence this non-invasive and widely available investigation has the potential to be used as an alternative to a fundoscopic screening of DR. This study aimed to determine the association of UACR with the presence of DR in patients with T2DM.

## Materials and methods

This retrospective study analyzed the data of patients who had visited the general medicine and ophthalmology out-patient departments (OPD) at a tertiary care hospital in South India between 2015 and 2018 for a regular follow-up of glycemic control. As part of their routine visit, all patients had undergone clinical assessment, biochemical and ophthalmological evaluation on an appointment basis.

Data, including the clinical history, known duration of T2DM, the presence of other non-communicable diseases, and medication details, was extracted from the medical records of all the patients. Educational qualifications and the details of physical activity recorded as per the global physical activity questionnaire were retrieved [12]. Physical examination details, including the values of their clinic blood pressure, height in meters (m), and body weight in kilograms (kg), was extracted. The body mass index (BMI) was derived using the formula BMI = weight (kg)/{height(m)}^2^. The fundoscopy findings were noted in accordance with the Early Treatment for Diabetic Retinopathy Study (ETDRS) classification to divide the patients into one out of five categories [13]:

i) No DR

ii) Mild non-proliferative diabetic retinopathy (NPDR): at least one microaneurysms; criteria not met for other levels of DR

iii) Moderate NPDR: microaneurysms or hemorrhages along with soft exudates, venous beading, and intra-retinal microvascular abnormalities

iv) Severe NPDR: microaneurysms or hemorrhages in all four quadrants, venous beading in two or more quadrants, intraretinal microvascular abnormalities in at least one quadrant

v) Proliferative diabetic retinopathy (PDR): neovascularization and/or vitreous or pre-retinal hemorrhage.

The reports of laboratory tests performed during the same OPD visit were extracted, including the values of fasting (FBS) and post-prandial blood glucose (PPBS) by the hexokinase method, glycated hemoglobin (HbA1) by high-performance liquid chromatography, and serum creatinine using Jaffe kinetic method. The estimated glomerular filtration rate (eGFR) was calculated using the Chronic Kidney Disease Epidemiology Collaboration (CKD-EPI) formula [14]. For women, creatinine <0.7 mg/dl eGFR = 144 × (creatinine/0.7)−0.329 × (0.993)age, and creatinine >0.7 mg/dl eGFR = 144 × (creatinine/0.7)−1.209 × (0.993)age; and for men, creatinine > 0.9 mg/dl eGFR = 141 × (creatinine/0.9)−1.209 × (0.993)age. 

The levels of albumin (automated turbidometry method) and creatinine (Jaffe kinase method) in the spot first-morning urine sample of the day of their visit that had been used to determine the UACR in the same visit was also extracted. Microalbuminuria was defined as a UACR between 30-300 mg/g and macroalbuminuria as UACR >300 mg/g [15].

All patients with T2DM over the age of 18 years were included in this study. Patients excluded from the study were those who had any of the following to be documented in them: past medical history of non-diabetic kidney disease; a history of concurrent fever, urinary tract infection or were detected to have urinary calculi at the time of assessment; microscopic or macroscopic hematuria; or corneal opacities, hyper-mature cataracts or other illnesses interfering with the interpretation of the direct ophthalmology findings.

Statistical analysis

Continuous data was represented as mean and standard deviation. Categorical data was represented in the form of frequencies and proportions. Data was entered into Microsoft Excel (Microsoft, Redmond, Washington) datasheet, and Statistical Package for Social Sciences version 20.0 (IBM Inc., Armonk, New York) was used to analyze the data. The Chi-square test or Fischer’s exact test (for 2x2 tables only) was used as a test of significance for qualitative data. An independent t-test was used as a test of significance to identify the mean difference between two quantitative variables. ANOVA was used as a test of significance to identify the mean difference between more than two quantitative variables. Following a univariate analysis of the predictor variables, a logistic multiple regression analysis was performed to identify the variables associated independently with the presence of diabetic retinopathy. The receiver operating characteristic (ROC) curve was plotted for the UACR values, and optimal cut-off points were chosen for the calculation of sensitivity, specificity, positive and negative predictive values in order to predict the association with diabetic retinopathy. An AUC of 0.5 would mean no discrimination, 0.7 to 0.8 would be considered to be acceptable, 0.8 to 0.9 considered excellent, and greater than 0.9 would be considered outstanding. a p-value of ≤0.05 was deemed to be statistically significant [16].

## Results

The medical records of 300 consecutive patients with T2DM who visited the general medicine OPD between 2015 and 2018 were accessed. Owing to the presence of one or more exclusion criteria, 28 were not considered for inclusion in the study. Finally, the data from a total of 272 patients was analyzed for this study. This included 156 male and 116 female patients. The average age of the patients included in the study was 60.35±10.56 years.

A total of 147/272 patients were diagnosed to have diabetic retinopathy, whereas the rest 125 had no retinopathy on fundus examination during the OPD visit. Among those with retinopathy, 135 patients were found to have NPDR (64 patients had mild, 53 patients had moderate, and 18 patients had severe), whereas 12 patients were diagnosed with PDR.

An incremental trend was seen with respect to the mean age (p<0.001) as well as the median duration of diabetes (p<0.001) among patients having DR (Table 1). On univariate analysis, a significantly higher number of retinopathy patients were found to have associated comorbidities such as hypertension, coronary artery disease (CAD), and hypothyroidism in comparison to those diabetics without retinopathy. The differences in their current pattern of antidiabetic medications and their physical activity levels were also found to be significant. There was also a statistically significant difference noticed with regards to the history of the presence of T2DM among immediate family members between the two groups.

**Table 1:** Univariate analysis of variables for association with DR *Age and eGFR presented as mean ± standard deviation, other variables are presented as median(inter-quartile range) NPDR - non-proliferative diabetic retinopathy, PDR - proliferative diabetic retinopathy, DM - diabetes mellitus, BMI - body mass index, eGFR - estimated glomerular filtration rate, FBS - fasting blood sugar, PPBS - post-prandial blood glucose, HbA1c - glycated hemoglobin, UACR - urinary albumin to creatinine ratio, PUC - pre-university degree, OAD - oral antidiabetic medication

	No DR (N=125)	Mild NPDR (N=64)	Moderate NPDR (N=53)	Severe NPDR (N=18)	PDR (N=12)	p-value
Age^*^ (years)	54.97±9.55	61.06±8.69	67.25±8.68	69.06±7.32	69.08±8.77	<0.001
Duration of DM (years)	5(2.5-8.5)	10(6-12)	15(10-21.5)	19(12-22.25)	20(15.3-23)	<0.001
BMI (Kg/m^2^)	27.34(25.4-29.5)	28.69(26.8-30.4)	28.2(25.9-30.74)	28.775(24.4-31.58)	30.18(24.08-32.29)	0.125
Gender						
Female	58 (46.4%)	23(35.9%)	21(39.6%)	7(38.9%)	58(46.4%)	0.492
Male	67(53.6%)	41(64.1%)	32(60.4%)	11(61.1%)	67(53.6%)	
Comorbidities						
Hypertension	44(35.2%)	34(53.1%)	33(62.3%)	16(88.9%)	8(66.7%)	<0.001
Coronary artery disease	7(5.6%)	8(12.5%)	21(39.6%)	8(44.4%)	6(50.0%)	<0.001
Hypothyroid	18(14.4%)	1(1.6%)	4(7.5%)	0(0%)	0(0%)	0.014
Family History of DM	48(38.4%)	34(53.1%)	26(49.1%)	8(44.4%)	9(75.0%)	0.075
Education						
Illiterate	22(17.6%)	14(21.9%)	11(20.8%)	2(11.1%)	22(17.6%)	0.589
Schooling	29(23.2%)	15(23.4%)	14(26.4%)	8(44.4%)	29(23.2%)	
PUC	12(9.6%)	4(6.2%)	6(11.3%)	0(0%)	12(9.6%)	
Graduation	52(41.6%)	24(37.5%)	19(35.8%)	7(38.9%)	52(41.6%)	
Masters	10(8.0%)	7(10.9%)	3(5.7%)	1(5.6%)	10(8.0%)	
Current treatment						
Not on treatment	2(1.6%)	0(0%)	0(0%)	0(0%)	0(0%)	0.04
Insulin	78(62.4%)	30(46.9%)	20(37.7%)	6(33.3%)	3(25.0%)	
OAD	14(11.2%)	11(17.2%)	8(15.1%)	3(16.7%)	6(50.0%)	
Insulin and OAD	31(24.8%)	23(35.9%)	25(47.2%)	9(50.0%)	3(25.0%)	
Activity						
Inactive	91(72.8%)	42(66.7%)	46(86.8%)	17(94.4%)	9(75.0%)	0.035
Active	34(27.2%)	21(33.3%)	7(13.2%)	1(5.6%)	3(25.0%)	
FBS (mg/dl)	182(161.5-216)	185(156-214.75)	184(152-216)	213.5(166.5-218.25)	162(136.5-213.15)	0.476
PPBS (mg/dl)	232(202-288.5)	232(202.5-283)	233(207-290)	274(225.75-329.5)	220(177.75-313.5)	0.267
HbA1C (%)	9.5(7.8-11.2)	9.25(7.8-11.05)	9.7(7.9-11.5)	10.8(9-12.9)	9.6(7.225-10.125)	0.115
eGFR^*^ (ml/min/1.73 m^2^)	74.95±26.09	56.06±24.20	56.90±29.41	49.33±32.78	54.41±43.12	<0.001
Uncontrolled FBS	13(48.1%)	8(29.6%)	3(11.1%)	1(3.7%)	2(7.4%)	0.631
Uncontrolled PPBS	13(43.3%)	9(30.0%)	5(16.7%)	0(0%)	3(10.0%)	0.248
Dyslipidemia	36(38.7%)	21(22.6%)	25(26.9%)	8(8.6%)	3(3.2%)	0.14
Microalbuminuria	56(64.4%)	16(18.4%)	12(13.8%)	1(1.1%)	2(2.3%)	<0.001

The UACR was found to be higher, and eGFR values were lower among those with DR. The median UACR value among patients with DR was found to be 214 (45.4-1260) mg/g, which was seen to be significantly higher than for those without retinopathy for whom it was 42.6 mg/g (18.21-183.3 mg/g) (p<0.001) (Table 2).

**Table 2:** UACR comparison between diabetics with and without retinopathy DR - diabetic retinopathy, UACR - urinary albumin to creatinine ratio

	No DR (mg/g)	DR (mg/g)	p-value
UACR median(interquartile range)	42.6(18.21-183.3)	214 (45.4-1260)	<0.001

However, on logistic regression analysis, only three variables had an independent association with the presence of DR, namely UACR, known duration of T2DM, and past history of coronary artery disease (Table 3).

**Table 3:** Logistic regression analysis table displaying the variables with statistically significant independent association with the presence of diabetic retinopathy DM - diabetes mellitus, CAD - coronary artery disease, UACR - urinary albumin to creatinine ratio

	Adjusted odds ratio	95% confidence intervals		p-value
		Lower	Upper	
Duration of DM	3.443	1.875	6.324	0
Presence of CAD	3.955	1.562	10.016	0.004
UACR >30 mg/g	2.803	1.502	5.234	0.001

185 patients were detected to have albuminuria (97 microalbuminuric and 88 microalbuminuria), while the rest (87 patients) were normoalbuminuric at the time of their hospital visit. The mean UACR values were observed to correlate with the increasing severity of DR, with the lowest values of UACR found in those with grade 1 NPDR (171.4[26.5-621.9]) mg/g while the set of patients with PDR had the highest UACR {1243.4[87.5-2189.25] mg/g).

**Figure 1:**
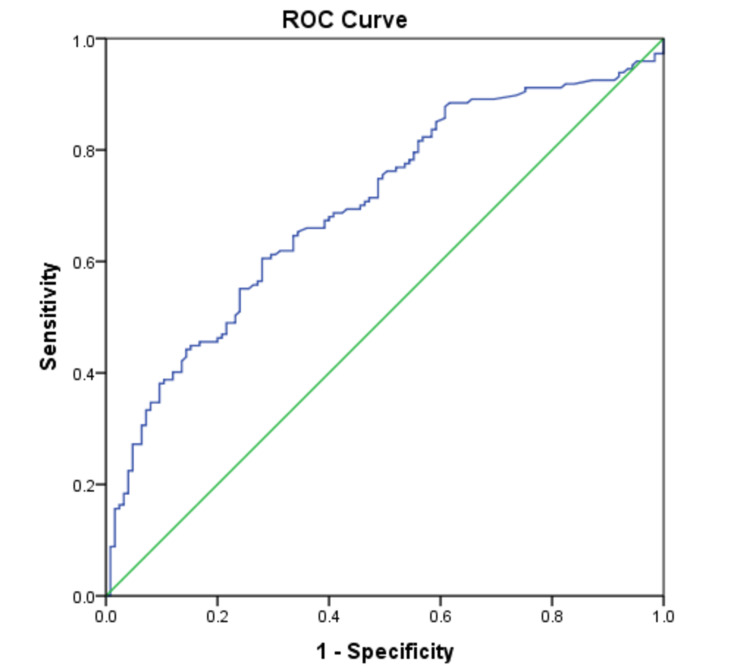
Receiver operating characteristic curve for the association of UACR with diabetic retinopathy ROC - receiver operating characteristic, UACR - urinary albumin creatinine ratio

The ROC curve for UACR in predicting the presence of DR (Figure 1) had an area under the curve of 0.70 (95% CI 0.638-0.762) (Table 4). On calculating the Youden index, a UACR cut off value of 140 mg/g was seen to have the best predictability for DR with a sensitivity of 60.5% and a specificity of 72%, along with positive (PLR) and negative likelihood ratios (NLR) of 2.16 and 0.54, respectively.

**Table 4:** Area under the receiver operating characteristic curve in Figure 1

Area under the curve	Std. Error^a^	Asymptotic Sig.^b^	Asymptotic 95% confidence interval	
			Lower bound	Upper bound
0.700	0.032	0.000	0.638	0.762

Further, the ROC curve was recalculated for the 137 patients in the study who were not on Angiotensin-converting enzyme (ACE) inhibitors and Angiotensin receptor blockers (ARB). The UACR cut-off value of 140 mg/g was found to have a comparable sensitivity (60%), specificity (79%), PLR (2.65), and NLR (0.24) to indicate the presence of DR in them just as it did for the entire study sample.

**Figure 2:**
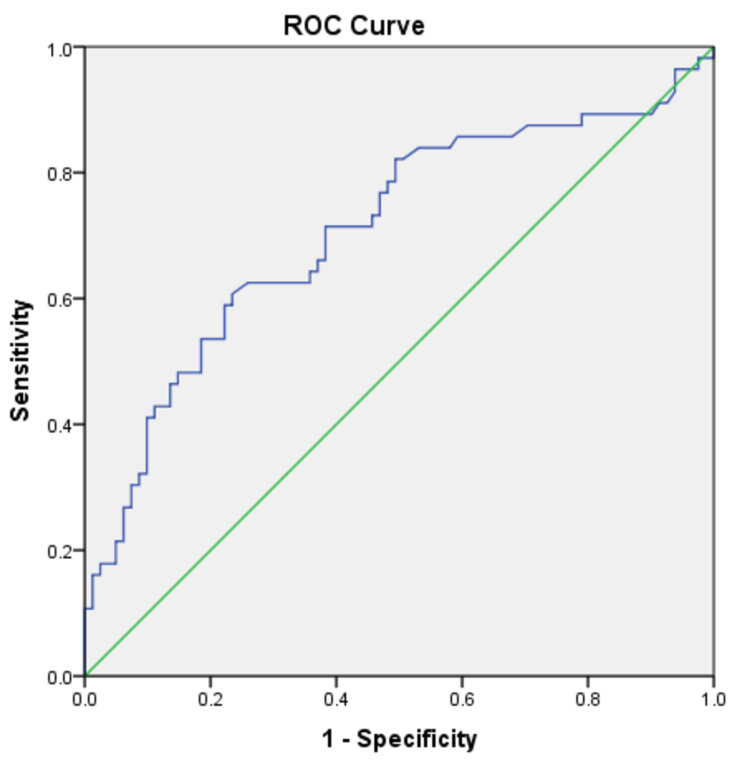
Receiver operating characteristic curve for the association of UACR with diabetic retinopathy for those not on ACE inhibitors or ARB ROC - receiver operating characteristic, UACR - urinary albumin creatinine ratio, ACE - angiotensin-converting enzyme, ARB - angiotensin receptor blockers

**Table 5:** Area under the receiver operating characteristic curve in Figure 2

Area under the curve	Std. Error^a^	Asymptotic Sig.^b^	Asymptotic 95% confidence interval	
			Lower bound	Upper bound
0.708	0.047	0.000	0.617	0.800

## Discussion

In the present retrospective study, it was observed that rising levels of UACR were independently associated with the severity of DR among patients with T2DM. Albuminuria was seen to be independently associated with the presence of DR in comparison to normoalbuminuria. UACR beyond 140 mg/g was observed to be strongly associated with the presence of DR with a sensitivity of 60.5% and a specificity of 72%. Hence, this suggests that the diabetics with a UACR less than 140 mg/g may have a lesser probability of having developed DR and may consider postponing their annual fundus examination until the period of peak COVID-19 outbreak passes and COVID-19 related lockdown restrictions are eased. This highlights the potential of UACR to be used as a screening tool to predict the presence of DR. ACE inhibitors and ARB have proven anti-proteinuric effects [17]. In order to eliminate the influence of ACE inhibitors and ARB, the data of diabetics without hypertension and CAD was reanalyzed, and it showed that the same cut-off value of 140 mg/g still performed comparably.

Past studies and systematic reviews have explored the role of albuminuria in predicting the presence of DR with inconsistent results [18-20]. Rani et al. had also demonstrated that patients with micro and macroalbuminuria were two and six times more likely to have DR, respectively, in comparison to those with normal urinary albumin excretion [20]. The cut-off value in our study is higher than those derived in certain other studies [21,22]. Potisat et al. failed to show an association between microalbuminuria and DR in their cross-sectional study [23]. But most of these studies have used either dipstick or 24-hour urinary estimation to determine the urinary albumin excretion rate, whereas relatively fewer studies have employed UACR for this purpose [21,24,25]. In the present study, although reduced eGFR was seen to be associated with DR on univariate analysis, this association was not evident on multivariate analysis. This is similar to the findings from other studies [26].

The association between DR and UACR is likely to be owing to the common underlying pathophysiological mechanisms involved in their development. Oxidative stress induced by chronic hyperglycemia, formation of advanced glycated end products, activation of protein kinase C pathway, abnormal activation of the renin-angiotensin-aldosterone system may all result in progression of DR and diabetic kidney disease (DKD) in parallel [24]. Endothelial dysfunction is considered to be the pathognomonic feature of the damage that occurs in the microcirculatory system within various organs in the body in T2DM. The extracellular matrix has anionic compounds like heparan sulfate proteoglycan. Enzymes involved in the metabolism of such structural components, such as the N deacetylase, are altered by hyperglycemia, thereby leading to endothelial dysfunction. Additionally, microalbuminuria and DR have several risk factors in common, including the known duration of diabetes and elevated blood pressure levels. Finally, DR may be accelerated in the presence of elevated fibrinogen and lipoproteins levels due to renal damage [19].

Although several variables in the present study were found to be associated with DR on univariate analysis, only three factors were found to be independently associated with the presence of DR, namely UACR, known duration of diabetes, and past history of CAD. Similar findings have been noted in earlier studies [22,27]. Often the duration of T2DM determined from patients’ history does not reflect the true duration of the disease since these patients may not develop symptoms for several years despite the presence of hyperglycemia [28]. T2DM is considered to be the equivalent of atherosclerotic coronary artery disease, yet patients with T2DM may develop silent CAD owing to autonomic dysfunction, which is common in long-standing diabetics. This may delay the detection of CAD [29]. Nevertheless, considering these other two factors in addition to UACR may enhance the effectiveness of DR screening. Developing an algorithm or a formula that incorporates all these parameters together may enhance the precision of DR screening.

Strengths of this study include the employment of robust exclusion criteria and the statistical methods in the analysis. The limitations include its retrospective design, limited study sample size, and that only a single value of UACR was available for all patients. The findings of this study will need to be confirmed using larger prospective trials.

## Conclusions

The presence of albuminuria significantly correlated with the presence of DR in patients with T2DM in this study. During the COVID-19 pandemic, when healthcare services have come under tremendous strain, UACR may be used as a screening tool in the detection of DR. A UACR value of greater than 140 mg/g may be considered as a cut-off to determine the need to undergo fundus examination for DR until the prevailing restrictions due to the pandemic are eased.
